# Widely Targeted Metabolomics of *Morchella Sextelata* After Hot‐Air Drying

**DOI:** 10.1002/fsn3.70826

**Published:** 2025-09-18

**Authors:** Jingfang Yi, Hongbo Guo, Zihan Cao, Ruiheng Yang, Ying Song, Ying Lin, Aiguo Xu, Xiaodan Yu

**Affiliations:** ^1^ Key Lab of Biological Resources and Biosecurity Institute of Plateau Biology of Xizang Autonomous Region Lhasa China; ^2^ College of Biological Science and Technology Shenyang Agricultural University Shenyang China; ^3^ College of Life Engineering Shenyang Institute of Technology Fushun China; ^4^ Institute of Edible Fungi Shanghai Academy of Agricultural Sciences Shanghai China; ^5^ Edible Fungi Research Institute Liaoning Academy of Agricultural Sciences Shenyang China

**Keywords:** flavoromics, hot‐air drying, *Morchella sextelata*, non‐volatile metabolite, volatile metabolite

## Abstract

*Morchella sextelata* is a valuable and popular commercial edible fungus in China. Hot‐air drying can enhance the flavor and quality of edible mushrooms; however, the resulting changes in volatile and non‐volatile metabolites have not been investigated for *M. sextelata*. In the present study, we used a widely targeted metabolomics approach combining ultra‐high‐performance liquid chromatography–tandem mass spectrometry (UHPLC–MS/MS) and gas chromatography–mass spectrometry (GC–MS) to explore the dynamic variations in metabolites after hot‐air drying and the influence of drying treatment on the flavoromics of different sections of the fruiting bodies of *M. sextelata*. In total, we identified 1419 non‐volatile and 270 volatile metabolites. Based on multivariate statistical analysis, we identified 511 metabolites with significant differences between fresh and dry products, revealing that hot‐air drying resulted in more nutty, toasty, and fatty flavors, particularly in the pileus. Moreover, hot‐air drying enriched the beneficial dietary components of the pileus. Through the elucidation of the metabolite changes that occur in *M. sextelata* after hot‐air drying, this study provides theoretical support for the higher commercial value of the pileus after drying and can be applied to improve the quality of dried morels.

## Graphical abstract

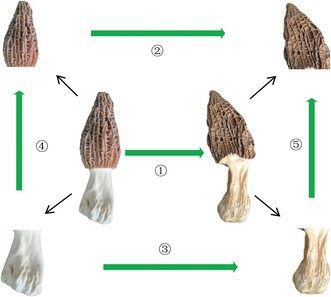

## 1 Introduction

Morel (*Morchella* spp.), which encompasses all species of the *Morchella* genus, is one of the most favored mushrooms worldwide because of its distinctive aroma, crisp texture, and delectable fruiting bodies (Du et al. 2015; Tietel and Masaphy 2018a). As well as being rich in amino acids, sugars, vitamins, minerals, and other nutrients (Dong et al. 2022), *Morchella* species contain polysaccharides, flavonoids, alkaloids, phenols, quinones, and other biologically active ingredients (Sunil and Xu 2022; Tietel and Masaphy 2018b), affording these mushrooms anti‐cancer; (Liu, Sun, et al. 2016); Haq et al. 2023), antioxidant (Li et al. 2022; Wang et al. 2020), and immunomodulatory activities (Li et al. 2021). Traditionally, commercially available morels were collected from wild resources; however, the application of exogenous nutrient bag technology has since led to the large‐scale cultivation of morels in China. With a production volume of 240,000 tons in 2021, China is currently the largest producer and exporter of *Morchella* spp.

However, owing to their crisp texture and high water content, fresh fruiting bodies are prone to browning, breakage, and decay, which seriously affect their economic value (Gao et al. 2022). Consequently, dehydration is commonly used to extend shelf life and enhance economic benefits, with dried products accounting for more than 90% of all morel sales. Hot‐air drying, which benefits from low costs, simple equipment, and easy operation, is widely used for dewatering and drying edible fungi, such as *Lentinus edodes* (Hu et al. 2021; Qin et al. 2020; Wen et al. 2022), *Pleurotus eryngii* (Tolera and Abera 2017), *Cordyceps militaris* (Zhang et al. 2022), *Oudemansiella raphanipes* (Shen et al. 2023), and *M. sextelata* (Li et al. 2023).

Hot‐air drying also plays a vital role in enhancing the unique flavor and quality of mushrooms. Straight‐chain and cyclic sulfur compounds contribute significantly to the distinctive flavor of *shiitake* mushrooms (Hiraide et al. 2004). Specifically, fresh *shiitake* mushrooms exhibit no straight‐chain sulfur compounds and few cyclic sulfur compounds, whereas mushrooms subjected to hot‐air drying produce more abundant and varied straight‐chain and cyclic sulfur compounds, resulting in a more intense mushroom flavor (Zhang et al. 2021). However, all drying processes lead to a decline in amino and organic acids (Zhang et al. 2021). (Shen et al. (2023)) studied the effects of different drying methods on the taste‐active compounds of *O. raphanipes* and determined that ultrasound‐assisted hot‐air drying at 60°C led to the highest content of free amino acids and equivalent umami concentration and helped preserve the organic acids, umami amino acids, sweet amino acids, and other flavor substances in *O. raphanipes*. Zhang et al. (2022) compared the effects of different drying methods on the flavor compounds of *C. militaris* and determined that hot‐air drying resulted in higher contents of umami amino acids, sweet amino acids, and aldehydes, which contributed to the overall aroma. Furthermore, Li et al. (2023) studied the characteristic fingerprints of the volatile flavor components of *M. sextelata* using different drying methods (freezing, hot‐air drying, and natural air drying) and showed that hot‐air drying promoted the formation of heterocyclic compounds and ketones such as 2‐cyclohexene‐1‐1, furan, and 3‐phenyl‐ through thermal reaction. However, as their study only compared the volatile characteristics of *M. sextelata* under different dehydration methods, changes in the non‐volatile metabolites of *M. sextelata* caused by hot‐air drying have not been determined, and volatile metabolite changes require more in‐depth investigation.

Metabolomics is an emerging tool used to study the final products of low‐molecular‐weight (< 1000 Da) gene expression in living organisms (Uawisetwathana and Karoonuthaisiri 2019). Widely targeted metabolomics combines the advantages of both targeted and non‐targeted metabolomics, providing high throughput, broad coverage, and high sensitivity (Wang et al. 2021). Metabolomics technology, through metabolic fingerprinting and multivariate statistical analysis, can not only accurately differentiate edible mushroom varieties and origins, but also identify critical targets for optimizing cultivation conditions and improving processing techniques. Furthermore, dynamic metabolic network analysis facilitates the understanding of environmental factors' influence on metabolite accumulation mechanisms, thereby establishing a theoretical foundation for quality control and value‐added utilization of edible mushroom resources. Currently, widely targeted metabolomic approaches have been successfully applied to food science fields such as food processing (Wang et al. 2021; Xiao et al. 2022), food storage (Shi et al. 2022), key component identification (Zou et al. 2020), and analysis of the evolutionary trajectory of non‐volatile metabolites. Therefore, in this study, we used a widely targeted metabolomics approach to determine the relationship between the non‐volatile and volatile metabolites of *M. sextelata* and their changes during hot‐air drying. Specifically, we combine ultra‐high‐performance liquid chromatography‐electrospray ionization‐tandem mass spectrometry (UPLC‐ESI‐MS/MS) and gas chromatography–mass spectrometry (GC–MS) to detect non‐volatile and volatile metabolites, respectively, in fresh and dried *M. sextelata*, thereby elucidating the effects of hot‐air drying on the flavoromics of *M. sextelata*. This study provides a scientific basis for improving the quality of dried morels.

## 2 Materials and Methods

### 2.1 Plant Material

Fresh fruiting bodies of *M. sextelata* (Mel‐6, the experimental strains were preserved in Shenyang Agricultural University), free from mechanical or insect damage, and were harvested on March 24, 2023, from Haifeng Village, Beipiao City, Chaoyang (latitude 41.8°N, longitude 120.8°E, altitude 200 m), Liaoning Province, China.

### 2.2 Experimental Design and Sample Preparation

To ensure uniform physical properties of the samples, we selected fresh fruiting bodies of similar sizes with intact surfaces. The whole fruiting bodies were cut into two parts, the pileus and the stipe, and dried by hot‐air drying. We then refrigerated all morels in the lower layer of the refrigerator (−20°C) before testing. Fresh morels were removed from the refrigerator and divided into the pileus (cap) and stipe (stem). Before the drying operation, the dryer was turned on and run for 30 min until a stable state was reached. We then weighed the pileus and stipe of fresh morels (135 ± 0.5 g each) and placed them on a stainless‐steel mesh tray. The samples were dried by hot air at an initial temperature of 45°C for 3 h, then at 50°C for 3 h, and finally at 60°C until dry, then weighed again. All drying experiments were performed three times, and the average values were used for analysis. Dry products were sealed and stored in a refrigerator at −20°C.

### 2.3 Sample Preparation and Extraction for Metabolomic Analysis

500 milligrams of powdered sample was transferred immediately to a 20‐mL headspace vial (Agilent, Palo Alto, CA, USA) containing a saturated NaCl solution. Vials were sealed using crimp‐top caps with TFE‐silicone headspace septa (Agilent Technologies). For solid‐phase microextraction, each vial was placed at 60°C for 5 min before exposing a 120‐μm DVB/CWR/PDMS fiber (Agilent) to the headspace of the sample for 15 min at 60°C.

We then performed vacuum freeze‐drying by placing the biological samples in a lyophilizer (Scientz‐100F) then grinding them to a powder (at 30 Hz for 1.5 min) using a grinder (MM 400, Retsch). Subsequently, we weighed 50 mg of sample powder using an electronic balance (MS105DΜ) and added 1200 μL of pre‐cooled (−20°C) 70% methanolic aqueous internal standard extract (for samples less than 50 mg, we added the extract at a rate of 1200 μL extractant per 50 mg sample). We then vortexed the samples once every 30 min for 30 s for a total of six times. After centrifugation (at a rotation speed of 12,000 rpm for 3 min), we aspirated the supernatant, filtered the samples through a microporous membrane (with a pore size of 0.22 μm), and stored them in injection vials for UPLC‐MS/MS analysis.

### 2.4 GC–MS Conditions

After sampling, the volatile organic compounds were desorbed from the fiber coating in the injection port of the GC apparatus (Model 8890; Agilent) at 250°C for 5 min in splitless mode. Volatile organic compounds were identified and quantified using an Agilent Model 8890 gas chromatograph and 7000D mass spectrometer (Agilent), equipped with a 30 m × 0.25 mm × 0.25 μm DB‐5MS (5% phenyl‐polymethylsiloxane) capillary column. Helium was used as the carrier gas at a linear velocity of 1.2 mL/min. The temperature of the injector and detector was maintained at 250°C and 280°C, respectively. The oven temperature program was as follows: 40°C for 3.5 min, increase at 10°C/min to 100°C, increase at 7°C/min to 180°C, increase at 25°C/min to 280°C, then held for 5 min. Mass spectra were recorded in electron impact ionization mode at 70 eV. The quadrupole mass detector, ion source, and transfer line temperatures were set to 150°C, 230°C, and 280°C, respectively. MS‐selected ion monitoring mode was used to identify and quantify the analytes.

### 2.5 UPLC Conditions

The sample extracts were analyzed using a UPLC‐ESI‐MS/MS system (UPLC, ExionLC AD, https://sciex.com.cn/) and a tandem mass spectrometry system (https://sciex.com.cn/). The analytical conditions were as follows: UPLC column, Agilent SB‐C18 (1.8 μm, 2.1 × 100 mm); mobile phase (solvent A and B), pure water with 0.1% formic acid and acetonitrile with 0.1% formic acid, respectively. Sample measurements were conducted using a gradient program with starting conditions of 95% A and 5% B. Within 9 min, a linear gradient of 5% A and 95% B was programmed; this composition was maintained for 1 min. Subsequently, the composition was adjusted to 95% A and 5% B within 1.1 min and maintained for 2.9 min. The flow velocity was set to 0.35 mL/min, the column oven was set to 40°C, and the injection volume was 2 μL. The effluent was alternately connected to an ESI‐triple quadrupole linear ion trap (QTRAP)‐MS system.

### 2.6 ESI‐QTRAP‐MS/MS

The ESI source operation parameters were as follows: source temperature 500°C; ion spray voltage (IS) 5500 V (positive ion mode)/−4500 V (negative ion mode); ion source gas I, gas II, and curtain gas were set to 50, 60, and 25 psi, respectively; high collision‐activated dissociation. QTRAP scans were acquired via multiple reaction mode (MRM) experiments with the collision gas (nitrogen) set to medium. The declustering potential and collision energy for individual MRM transitions were determined with further optimization. A specific set of MRM transitions was monitored for each period based on the metabolites eluted within this period.

### 2.7 Multivariate Data Analysis and Statistical Analysis

#### 2.7.1 Principal Component Analysis

Principal Component Analysis (PCA) is an unsupervised pattern recognition method for multivariate statistical analysis. It employs orthogonal transformation to convert a set of potentially correlated variables into a group of linearly uncorrelated variables, termed principal components. Unsupervised principal component analysis (PCA) was performed using the prcomp statistical function in R (www.r‐project.org). The data were scaled to unit variance prior to unsupervised PCA.

#### 2.7.2 Hierarchical Cluster Analysis and Pearson Correlation Coefficients

Cluster Analysis is a multivariate statistical method for classification that groups individuals, objects, or subjects based on their characteristics. The objective is to maximize homogeneity within each cluster while ensuring maximal heterogeneity between different clusters. Hierarchical cluster analysis of samples and metabolites was performed, and the results were presented as heatmaps with dendrograms. Pearson correlation coefficients between samples were calculated using the cor function in R and presented only as heatmaps. Both analyses were conducted using the R package Complex Heatmap. For hierarchical cluster analysis, the normalized signal intensities of the metabolites (unit variance scaling) were visualized as a color spectrum.

#### 2.7.3 Identification of Differential Metabolites

Orthogonal Projections to Latent Structures Discriminant Analysis (OPLS–DA) integrates Orthogonal Signal Correction (OSC) with Partial Least Squares Discriminant Analysis (PLS–DA) methodology. This approach decomposes the X–matrix information into two distinct components: Y–correlated variations and Y–orthogonal variations. By systematically removing the orthogonal (uncorrelated) variations, OPLS–DA enables more effective screening of discriminant variables. For two‐group analysis, differential metabolites were determined by absolute Log_2_ fold change values ≥ 1 and variable importance in projection values > 1, the latter of which were extracted from the orthogonal partial least squares discriminant analysis (OPLS–DA) results, which also contained score plots and permutation plots, and were generated using the R package MetaboAnalystR. The data was log−transformed (log_2_) and mean‐centered prior to OPLS2013DA. A permutation test (200 permutations) was performed to avoid overfitting.

#### 2.7.4 Kyoto Encyclopedia of Genes and Genomes (KEGG) Annotation and Enrichment Analysis

Identified metabolites were annotated using the KEGG COMPOUND database (http://www.kegg.jp/kegg/compound/) and annotated metabolites were mapped to the KEGG PATHWAY database (http://www.kegg.jp/kegg/pathway.html). Pathways with significantly mapped regulated metabolites were then used for metabolite set enrichment analysis, and their significance was determined based on the *p*‐values of the hypergeometric test.

## 3 Results

**FIGURE 1:**
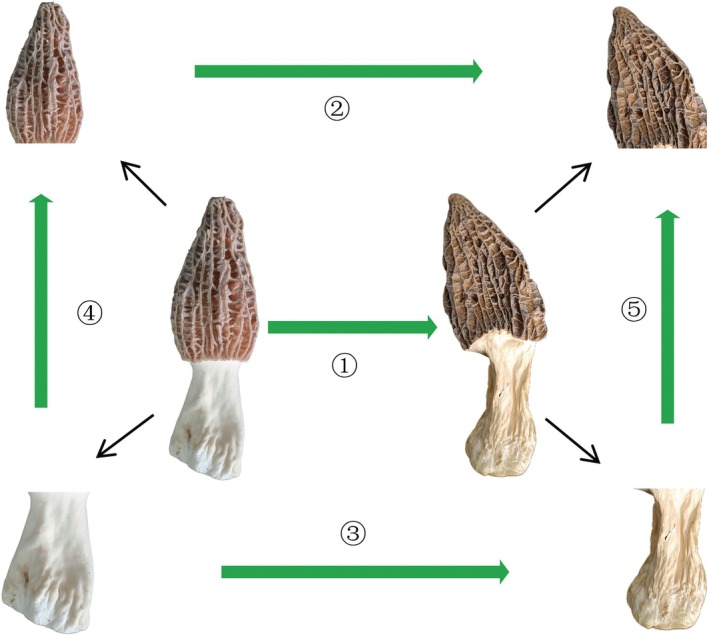
Summary diagram of metabolite analysis of *Morchella* before and after drying. ① To compare the changes of metabolites between fresh and hot air‐dried morels; for details, see 3.1. ② The changes of metabolites between fresh and dry fungus pileus were compared; for details, see manuscript 3.2.1. ③ The changes of metabolites between fresh and dried stipes were compared. For details, see manuscript 3.2.2. ④ Compare the changes of metabolites between fresh fungus pileus and fresh fungus stipe; for details, see manuscript 3.3.1. ⑤ Compare the changes of metabolites between dried fungus pileus and dried fungus stipe; for details, see manuscript 3.3.1. The arrow points to an overall upward trend in metabolite content.

In this study, a widely targeted metabolomics approach based on UPLC‐MS/MS combined with GC–MS was used to investigate alterations in non‐volatile and volatile metabolites during this process. The OPLS‐DA model was used to determine significant differences in non‐volatile and volatile metabolites between fresh and dried morels (Figure 1).

### 3.1 Metabolite Composition of *M. Sextelata* Before and After Drying

**FIGURE 2:**
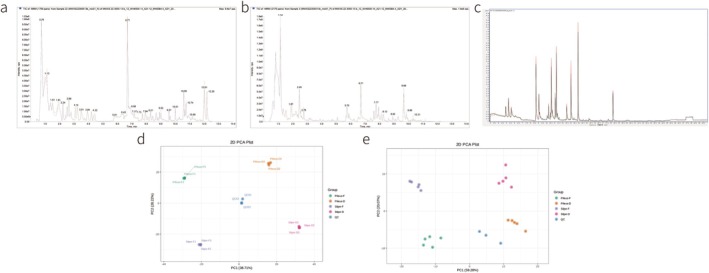
Total ion flow diagram of mixed quality control sample (TIC) and principal component analysis (PCA). (2a–2c) Non‐volatile metabolites QC sample total ion flow (TIC) diagram superposition diagram and principal component analysis (PCA) diagram; (2d, 2e) Volatile metabolites QC sample total ion flow (TIC) diagram superposition diagram and principal component analysis (PCA) diagram. PC1 represents the first principal component, PC2 represents the second principal component, PC3 represents the third principal component, and percentage represents the interpretation rate of this principal component to the data set. Each point in the diagram represents a sample, and samples in the same group are represented by the same color, and group is the grouping.

A widely targeted metabolomic approach was used to monitor fresh and dried morels. Quality control (QC) samples (a mixture of 50 μL of each sample, in triplicate) were analyzed to evaluate the reliability of the method. The total ion flow patterns of QC samples under positive and negative ionization modes were superimposed and demonstrated excellent repeatability (Figure 2). The total ion flow curve detected by metabolites exhibited high overlap; that is, the retention time and peak intensity were consistent, which indicates good MS signal stability when detecting the same sample at different times. The QC and test samples were further analyzed using an unsupervised PCA model based on the non‐volatile and volatile metabolites detected in this study. The four groups of morels were distinct, and the QC sample was positioned at the center of all test samples. This validates the reliability of the proposed method and confirms that the four *Morchella* species contained different metabolites (Figure 2).

A total of 1419 non‐volatile metabolites (Table S1) and 270 volatile metabolites (Table S2) were detected using UPLC‐MS/MS and GC–MS detection platforms, along with a self‐built database. The non‐volatile metabolites included 255 amino acids and their derivatives, 119 phenolic acids, 91 nucleotides and their derivatives, 60 flavonoids, 19 lignans and coumarins, one tannin (proanthocyanidins), 198 alkaloids, 121 terpenoids, 140 organic acids, 193 lipids, 17 quinones, one steroid, and 204 other metabolites (Figure S1a). The volatile metabolites included 52 heterocyclic compounds, 50 esters, 41 terpenoids, 31 alcohols, 25 hydrocarbons, 21 ketones, 17 aldehydes, 13 aromatics, six phenols, five acids, four nitrogen compounds, three amines, and two sulfur compounds (Figure S1b).

After hot‐air drying, the metabolites of the morels underwent significant changes. Drying added 37 new metabolites to the pileus, of which 21 were non‐volatile and 16 were volatile. Conversely, drying decreased eight metabolites in the stipe, all of which were non‐volatile, and added 35 metabolites, including 20 non‐volatile and 15 volatile substances. Overall, 19 types of metabolites increased after hot‐air drying, encompassing 15 volatile metabolites and four non‐volatile metabolites.

### 3.2 Significant Differences in Metabolite profiles and Flavoromics of *M. Sextelata* Before and After Drying

Comparative metabolomic analysis between four distinct sample groups—dried pileus vs. fresh pileus, dried stipe vs. fresh stipe, dried pileus vs. dried stipe, and fresh pileus vs. fresh stipe of *Morchella sextelata*—identified 920 significantly differential non‐volatile metabolites (Table S3) and 175 significantly differential volatile metabolites (Table S4). The detailed metabolite profiles across comparison groups are presented in Tables. A comparison of the metabolites in fresh and dried *M. sextelata* revealed 511 metabolites with significant differences between the two, including 420 non‐volatile metabolites and 91 volatile metabolites. In the pileus, 659 metabolites showed significant differences before and after drying, including 528 non‐volatile and 131 volatile metabolites. Among the non‐volatile metabolites, 431 were significantly upregulated and 97 were significantly downregulated (Table S5). Among the volatile metabolites, 31 were significantly upregulated and 100 were significantly downregulated (Table S6). In the stipe, 712 metabolites showed significant differences before and after drying, including 592 non‐volatile and 120 volatile metabolites. Among the non‐volatile metabolites, 516 were significantly upregulated and 76 were significantly downregulated (Table S5). Among the volatile metabolites, 40 were significantly upregulated and 80 were significantly downregulated (Table S6). Based on the Venn diagram, 108 non‐volatile and 40 volatile metabolites were observed only in the pileus, whereas 172 non‐volatile and 29 volatile metabolites were noted only in the stipe (Figure S2a,b). Based on flavoromics analysis, the sensory flavors green, fruity, sweet, herbal, floral, and earthy were weakened after drying by the downregulation of related metabolites. In contrast, nutty, roasted, and fatty sensory flavors were enhanced by the upregulation of related metabolites.

#### 3.2.1 Comparison of Significantly Different Metabolites in the Pileus of *M. Sextelata* Before and After Drying

**FIGURE 3:**
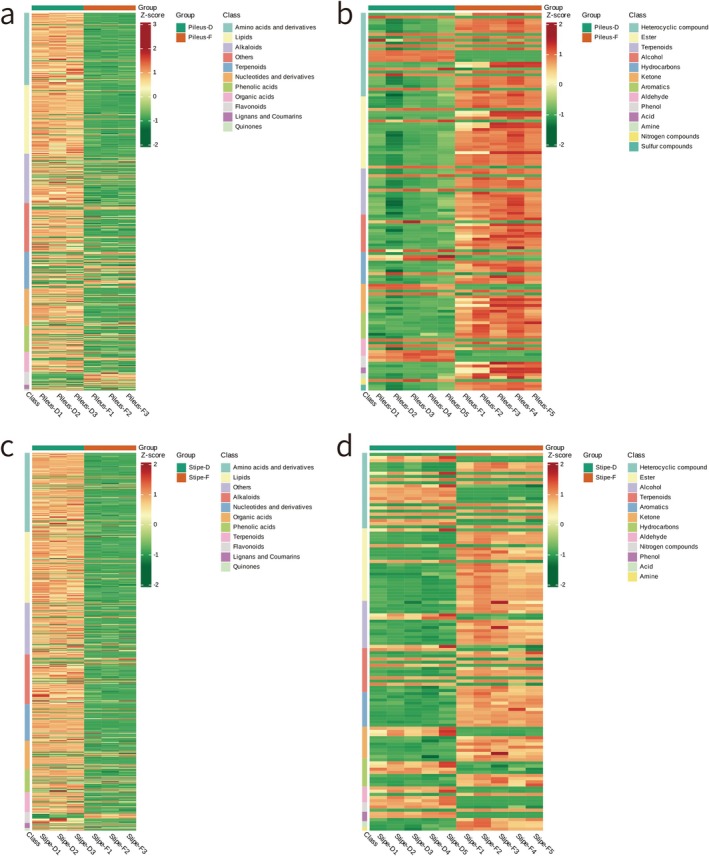
Differential metabolite clustering heat map. (3a) Cluster heat map of non‐volatile metabolites with significant difference before and after the pileus drying; (3b) cluster heat map of volatile metabolites with significant difference before and after pileus drying; (3c) cluster heat map of non‐volatile metabolites with significant difference before and after stipe drying; (3d) cluster heat map of volatile metabolites with significant difference before and after stipe drying. Horizontal is the sample name, vertical is the differential metabolite information, group is the group, and different colors are the colors filled with different values obtained after standardized treatment of different relative contents (red represents high content, green represents low content). Where class is the first class classification of substances; on the right side of the chart, the colors correspond to the metabolite species.

Among the non‐volatile metabolites with significant differences in the pileus after drying, the relative contents of amino acids and derivatives, lipids, alkaloids, nucleotides and derivatives, phenolic acids, organic acids, and quinones were increased, terpenoid metabolites remained unchanged, and the relative flavonoid, lignan, and coumarin contents were decreased (Figure 3a). The relative contents of esters, terpenoids, alcohols, hydrocarbons, ketones, aromatics, acids, amines, and sulfur compounds were also decreased. Heterocyclic compounds and phenol metabolites remained unchanged, whereas the relative contents of aldehydes and nitrogen compounds were increased (Figure 3b).

Next, we analyzed the top 20 non‐volatile and volatile metabolites with significant differences in the pileus after drying. Eighteen non‐volatile metabolites were significantly upregulated and two were significantly downregulated. Sixteen volatile metabolites were significantly upregulated and four were significantly downregulated (Figure S3a,b). Amino acids, fatty acids, terpenoids, and alkaloids were the primary non‐volatile metabolites in the pileus with significant differences after hot‐air drying, whereas the volatile metabolites were predominantly heterocyclic pyrazines.

#### 3.2.2 Comparison of Significantly Different Metabolites in the Stipe of *M. Sextelata* Before and After Drying

Among the non‐volatile metabolites with significant differences in the stipe after drying, only flavonoid metabolites were downregulated, whereas the relative contents of the other ten metabolites were increased (Figure 3c). Among the volatile metabolites with significant differences, the relative contents of esters, alcohols, terpenoids, aromatics, ketones, acids, and amines were decreased, heterocyclic compounds and hydrocarbon metabolites remained relatively unchanged, and the relative contents of aldehydes, phenols, and nitrogen compounds were increased (Figure 3d).

Among the top 20 non‐volatile and volatile substances with significant differences after hot‐air drying, 17 non‐volatile metabolites were significantly upregulated, and three were significantly downregulated. Seventeen volatile metabolites were significantly upregulated, and three were significantly downregulated (Figure S3c,d). Terpenoids, alkaloids, and flavonoids were the primary non‐volatile metabolites with significant differences after hot‐air drying, whereas the dominant volatile metabolites were heterocyclic pyrazines and aldehydes.

#### 3.2.3 Comparison of Flavoromics of *M. Sextelata* Before and After Drying

**FIGURE 4:**
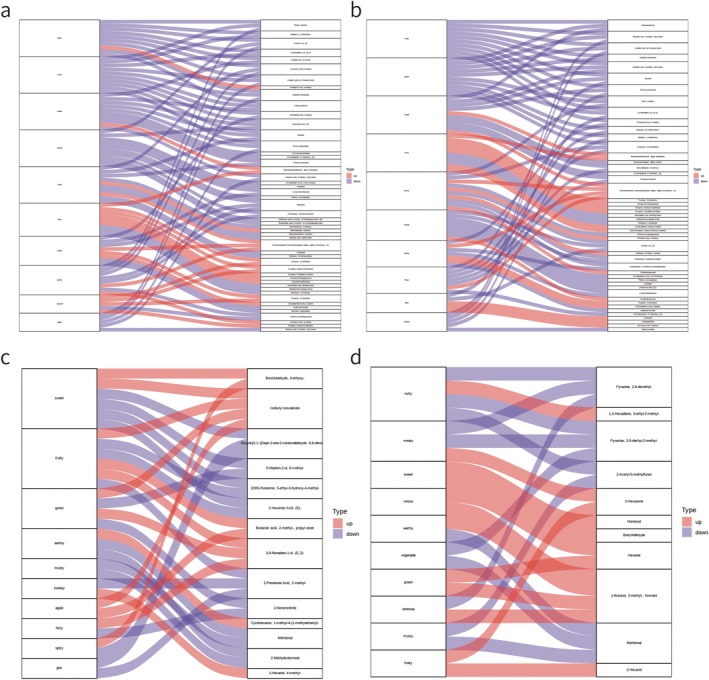
Sankey diagram of flavor omics. (4a) Sankey diagram of non‐volatile metabolites with significant difference before and after the pileus drying; (4b) Sankey diagram of volatile metabolites with significant difference before and after pileus drying; (4c) Sankey diagram of non‐volatile metabolites with significant difference before and after stipe drying; (4d) Sankey diagram of volatile metabolites with significant difference before and after stipe drying; the left column represents sensory flavor characteristics, the right column represents differential metabolites, the red flow line represents upregulated (up) differential metabolite flow, and the blue flow line represents downregulated (down) differential metabolite flow. The height of the box in the left column depends on the number of differential metabolites displayed in the right column; the higher the number, the higher the box. When the sensory flavor characteristic corresponds to more than 10 differential metabolites, the top 10 differential metabolites with the largest VIP value are displayed.

After hot‐air drying, the individual flavors of *M. sextelata* exhibited significant changes. Based on the differential metabolites identified based on the screening criteria and the sensory flavor characteristics annotated in each difference comparison group, the top ten sensory flavors with the highest number of annotations were selected to draw a Sankey map (Figure 4). Compared with the fresh pileus, the flavor characteristics in the dried pileus were green, fruity, sweet, herbal, floral, nutty, woody, earthy, roasted, and apple (Figure 4a). Compared with the fresh stipe, the flavor characteristics of the dried stipe were fruity, green, sweet, nutty, earthy, herbal, woody, floral, fatty, and tropical (Figure 4b). The observed changes in flavor characteristics were primarily attributed to the upregulation and downregulation of metabolites; however, a change in one flavor characteristic may be related to changes in several metabolites, and changes in a single metabolite may also cause changes in multiple flavor characteristics.

### 3.3 Comparison of Metabolites and Flavoromics Between the Pileus and Stipe

Among the metabolites with significant differences between the fresh pileus and stipe, 441 were non‐volatile (338 downregulated and 103 upregulated metabolites) (Table S1) and 33 were volatile (18 downregulated and 15 upregulated metabolites) (Table S2). Among the metabolites with significant differences between the dried pileus and stipe, 372 were non‐volatile (210 downregulated metabolites and 162 upregulated metabolites) (Table S1) and 23 were volatile (6 downregulated metabolites and 17 upregulated metabolites) (Table S2). Among the differential non‐volatile metabolites between the fresh pileus and stipe, three times more were downregulated than upregulated, whereas no significant differences were observed between volatile upregulated and downregulated metabolites. In contrast, among the differential metabolites between the dry stipe and pileus, no significant differences were observed between non‐volatile upregulated and downregulated metabolites, whereas three times more volatile metabolites were upregulated than downregulated (Figure S2c,d). Based on the flavoromics analysis, the fresh pileus showed stronger sweet, earthy, musty, and jam flavors. Conversely, the dried pileus showed stronger nutty, meaty, vegetable, and musty flavors, whereas the dried stalk showed stronger sweet, vinous, green, and fruity flavors.

#### 3.3.1 Comparison of Significantly Different Metabolites Between the Pileus and Stipe Before Drying

The relative contents of lipids, alkaloids, nucleotides and derivatives, phenolic acids, organic acids, quinones, terpenoids, flavonoids, lignans, and coumarins, all non‐volatile metabolites with significant differences, were higher in the fresh pileus than in the fresh stipe. Conversely, steroid metabolites were observed in higher amounts in the stipe than in the pileus. Amino acids and their derivatives were uniformly distributed in the pileus and stipe (Figure S4a). The relative contents of ester, terpenoid, acid, and nitrogen compounds, all volatile metabolites with significant differences, were higher in the pileus than in the stipe. Conversely, alcohol, heterocyclic compounds, and hydrocarbons were higher in the stipe than in the pileus. The relative amounts of aldehyde and ketone metabolites were similar between the pileus and stipe (Figure S4b).

Among the top 20 significant non‐volatile metabolites, eight were higher in the stipes than in the pileus, whereas 12 were lower in the stipes than in the pileus (Figure S5a). Among the top 20 significant volatile metabolites, eight were higher in the stipes than in the pileus, and 12 were lower in the stipes than in the pileus (Figure S5b). The dominant non‐volatile metabolites that showed significant differences between fresh stipes and fresh pileus were phenolic acids, amino acids and their derivatives, alkaloids, and flavonoids, whereas the dominant volatile metabolites were heterocyclic esters and terpenoids.

#### 3.3.2 Comparison of Significantly Different Metabolites Between the Pileus and Stipe After Drying

The relative contents of alkaloids, phenolic acids, organic acids, quinones, and terpenoids, all non‐volatile metabolites, were higher in the dried pileus than in the dried stipe, whereas those of amino acids and their derivatives, lipids, nucleotides and their derivatives, flavonoids, lignans, and coumarins were higher in the stipe than in the pileus (Figure S4c). Among the volatile metabolites with significant differences, heterocyclic compounds, aldehydes, alcohols, esters, hydrocarbons, phenols, and terpenoids were higher in the stipe than in the pileus, whereas the relative amounts of ketone substances were similar between the pileus and stipe (Figure S4d).

Among the top 20 significantly different non‐volatile metabolites, four showed higher relative contents in the stipe than in the pileus, whereas 16 showed lower relative contents in the stipe than in the pileus (Figure S5c). Among the top 20 significantly different volatile metabolites, 14 showed higher relative contents in the stipe than in the pileus, whereas six showed lower relative contents in the stipe than in the pileus. The dominant non‐volatile metabolites with significant differences between dried stipes and dried pileus were flavonoids, phenolic acids, alkaloids, and flavonoids, whereas the dominant volatile metabolites were heterocyclic compounds, ketones, and aldehydes (Figure S5d).

#### 3.3.3 Comparison of Flavoromics Between the Pileus and Stipe

The fresh stipe is light gray to white, the pileus has a clear outline, and the meat is thick and elastic with a strong bacterial aroma. The dried pileus is highly regarded as a nutritional supplement and sold at high prices; however, the stipe is frequently discarded or sold at low prices. In this study, we analyzed the flavoromics of this phenomenon and discovered higher contents of metabolites such as benzaldehyde, 4‐ethoxy‐, isobutyl isovalerate, butanoic acid, 2‐methyl‐, propyl ester, 3,6‐nonadien‐1‐ol, (E,Z)‐, cyclohexanol, 1‐methyl‐4‐(1‐methylethenyl)‐, and 1‐hexanol, 4‐methyl‐ in the stipe compared to the pileus, as well as lower contents of metabolites such as Bicyclo[3.1.1]hept‐2‐ene‐2‐carboxaldehyde, 6,6‐dimethyl‐ 5‐hepten‐2‐ol, 6‐methyl‐ 2 (5 h)‐furanone, 5‐ethyl‐3‐hydroxy‐4‐methyl‐ 2‐hexenoic acid, (E)‐, 2‐pentenoic acid, 2‐methyl‐ 2‐nonenenitrile, methional, and 2‐methylisoborneol. This led to the fresh stipe exhibiting weaker sweet, earthy, musty, and jam flavors than the pileus. Because the contents of pyrazine, 2,6‐dimethyl‐, pyrazine, 3,5‐diethyl‐2‐methyl‐, 2‐acetyl‐5‐methylfuran, and methional were lower in the stipe, and the contents of 1,3‐hexadiene, 3‐ethyl‐2‐methyl‐, 2‐hexanone, hotrienol, benzaldehyde, hexanal, 2‐hexanol, and 1‐butanol, 3‐methyl‐, formate were higher in the pileus, the dried stipe showed less prominent nutty, meaty, vegetable, and musty flavors but more prominent sweet, vinous, green, and fruity flavors (Figure 4c,d).

## 4 Discussion

Owing to its advantages of large throughput, wide coverage, and high sensitivity, we adopted a widely targeted metabolomics approach combining UPLC‐MS/MS with GC–MS to identify changes in the non‐volatile and volatile metabolites of *M. sextelata* after hot‐air drying and explore their contribution to the sensory properties of *M. sextelata*. A total of 1419 non‐volatile metabolites and 270 volatile metabolites were identified in the fresh and hot‐air‐dried samples; 528 non‐volatile and 131 volatile metabolites showed significant differences before and after partial drying in the pileus, whereas 592 non‐volatile and 120 volatile metabolites showed significant differences before and after partial drying in the stipe. These changes contributed to enhanced nutty, roasted, and fatty flavors but diminished green, fruity, sweet, herbal, floral, and earthy flavors after hot‐air drying. Differences in metabolites and sensory flavors between the pileus and stipe were also analyzed; 441 non‐volatile and 33 volatile metabolites were significantly different between the fresh pileus and fresh stipe, whereas 372 non‐volatile and 23 volatile metabolites were significantly different between the dried pileus and dried stipe. The changes in non‐volatile metabolites (e.g., amino acids, lipids, terpenes, flavonoids, and alkaloids) and volatile metabolites (e.g., pyrazines, aldehydes, and furans) led to more intense sensory flavors of sweet, earthy, musty, and jam in the fresh pileus than in the fresh stipe, as well as stronger sensory flavors of nutty, meaty, vegetable, and musty in the dried pileus than in the dried stipe.

### 4.1 Influence of Hot‐Air Drying on the Commercial Value of *M. Sextelata*

In this study, we identified 511 metabolites with significant differences between dry and fresh products, including 420 non‐volatile and 91 volatile metabolites. Regarding non‐volatile substances, the dominant metabolites with significant changes were amino acids, fatty acids, alkaloids, terpenes, and flavonoids. There were several reasons for this observation. First, heat treatment degrades proteins in the mushroom fruiting body into amino acids (Wang et al. 2011). Second, at the beginning of hot‐air drying, the temperature activates synthase activity, promoting protein degradation and amino acid metabolism (Nooshkam et al. 2019). Among the metabolites of *M. sextelata* with significant changes, nootkatol, β‐ionone, phloroglucinol, 1,3,5‐benzenetriol, and 13‐KODE, which can inhibit photoaging (Woo et al. 2021). Non‐volatile metabolites that were significantly upregulated after hot‐air drying, such as (9Z,11E) 13‐OxooCTADECA‐9, 11‐Dienoic acid, and decumbic acid, enhance antioxidative (Zhou et al. 2016), anti‐cancer (Liu et al. 2004; Matsui et al. 2022), and antibacterial functions (Ko et al. 2022). Moreover, phenol and 2,6‐Octadienoic acid, 3,7‐dimethyl‐, methyl ester, which can be used as fragrances and preservatives (Brancato 1982; Hong et al. 2005), were significantly upregulated after drying, as was genipin, which increases the texture, gel strength, stability, and shelf life of food (Ahmed et al. 2023). The relative salicylamide content was significantly downregulated after drying, which inhibited the production of melanin (Ito and Sato 2021). Thus, the significant upregulation of these non‐volatile metabolites enhanced the shelf life of morels, enabling the long‐term storage and transportation of dried morels.

The flavors of edible fungi are primarily derived from flavor and odor compounds (Maga 1981). Flavor compounds in mushrooms include volatile organic compounds such as alcohols, aldehydes, ketones, and heterocycles. Among these, eight carbon‐containing (C8) compounds such as 1‐octene‐3‐ol, 3‐octanol, and 3‐octanone are the primary sources of mushroom odor (Aisala et al. 2019; Du et al. 2021; Guo et al. 2018; Sun et al. 2020; Zhang et al. 2018). Based on Tietel and Masaphy (2018a, 2022), the aroma volatility spectrum of morel mushrooms is similar to that of other mushrooms but with some unique characteristics and exhibits significant variability depending on the species and ripeness. In terms of volatile substances, the metabolites with significant changes were predominantly heterocyclic aldehydes and pyrazines. Among the top 15 volatile organic compounds in *M. sextelata*, ten were key flavor substances with unique characteristics, with pyrazines accounting for the largest proportion. Pyrazine is an important volatile Maillard reaction product formed primarily through Strecker degradation between α‐dicarbonyl compounds and amino acids (Yu et al. 2021). Both the metabolites of edible fungi after drying and the flavors caused by these metabolites undergo significant changes. In the case of *Boletus edulis* (Zhang et al. 2018), dehydration provided a more desirable roasted and flavored flavor and a less grassy and earthy flavor than fresh samples. This result is highly consistent with the nutty and baked flavors of dried morel mushroom products (Davis and Dean 2016). In this study, hot‐air drying resulted in morels with more nutty, roasted, and fatty flavors.

### 4.2 Influence of Hot‐Air Drying on the Commercial Value of the Pileus and Stipe of *M. Sextelata*

Pre‐cutting treatment plays a critical role in the drying behavior and quality of dried products by altering the shape parameters of the materials (Li et al. 2019; Mulet et al. 2000). Erenturk et al. (2005) reported that cutting rosehips accelerated the drying process and increased the retention of vitamin C compared to whole rosehips. Defraeye (2017) reported that different cutting sizes and shapes of fruits significantly affected the drying time and quality. However, a systematic evaluation of the effects of different cutting methods on the flavor and metabolites of dried mushroom products of a single size or shape has not previously been reported. Our study provides theoretical support for such research and holds important reference value. Notably, 441 non‐volatile and 33 volatile metabolites were significantly different between the fresh pileus and stipe, whereas 372 non‐volatile and 23 volatile metabolites were significantly different between the dried pileus and stipe.

In terms of non‐volatile metabolites with significant differences between the fresh stipe and pileus, in addition to the anti‐inflammatory, antioxidant, and anti‐tumor functions of each part, the pileus contains more nootkatol, genipin, and 2‐hydroxy‐3‐phenylpropanoic acid, which inhibits photoaging and bacteria, enhances the texture of mushrooms, and extends shelf life. Based on previous metabolite analyses of mushroom parts after drying, L‐aspartic acid increases saltiness and inhibits bitterness (Kim et al. 2014), whereas ethyl linoleate* inhibits melanin production (Huang et al. 2021). The relative contents of these two substances were significantly upregulated in the stipe of *M. sextelata*. N‐Acetyl‐l‐threonine is an important dietary component (Mortel et al. 2010), whose relative content was significantly higher in the pileus than in the stipe. In terms of volatile metabolites with significant differences, pyrazines in heterocyclic substances are products of the Maillard reaction and important aroma components (Dang et al. 2016), whose relative contents after drying were significantly higher in the pileus than in the stipe. Changes in volatile substances have an impact on flavor. In terms of sensory flavor, fresh stipes had less sweet, earthy, musty, and jam flavors than fresh pileus. Compared to dried stipe, dried pileus had weaker nutty, meaty, vegetable, and musty flavors and enhanced sweet, vinous, green, and fruity flavors.

In summary, compared with fresh stipe, fresh pileus showed a longer storage time, somewhat delayed deterioration, and a richer mushroom aroma. After hot‐air drying, the pileus contained more dietary components and exhibited a stronger toasted, nutty, and meaty aroma. These metabolomic and flavoromic characteristics explain the greater popularity and higher price of the pileus of *M. sextelata*.

## 5 Conclusion

This study systematically investigated the metabolic variations and flavor dynamics between fresh and dried tissues of *Morchella sextelata* fruiting bodies. Four experimental groups were established: fresh pileus (PF), dried pileus (PD), fresh stipe (SF), and dried stipe (SD). Utilizing advanced analytical platforms and a customized metabolite database, we identified 1419 non‐volatile and 270 volatile metabolites across all comparison groups. Significant differential metabolites included 920 non‐volatile and 175 volatile compounds. Fresh vs. hot‐air‐dried whole fruiting bodies: 420 non‐volatile and 91 volatile differential metabolites. PF vs. PD: 528 non‐volatile and 131 volatile differential metabolites. SF vs. SD: 592 non‐volatile and 120 volatile differential metabolites. PF vs. SF: 441 non‐volatile and 33 volatile differential metabolites. PD vs. SD: 372 non‐volatile and 23 volatile differential metabolites. Sensory and flavor profile analysis across comparison groups revealed significant alterations attributable to metabolite compositional changes. Based on flavoromics analysis, the sensory flavors green, fruity, sweet, herbal, floral, and earthy were weakened after drying by the downregulation of related metabolites. In contrast, nutty, roasted, and fatty sensory flavors were enhanced by the upregulation of related metabolites. Based on the flavoromics analysis, the fresh pileus showed stronger sweet, earthy, musty, and jam flavors. Conversely, the dried pileus showed stronger nutty, meaty, vegetable, and musty flavors.

## Author Contributions

**Xiaodan Yu:** project administration (equal), supervision (equal). **Jingfang Yi:** conceptualization (equal), resources (equal), software (equal), validation (equal), writing – original draft (equal), writing – review and editing (equal). **Hongbo Guo:** conceptualization (equal), formal analysis (equal), funding acquisition (equal), methodology (equal). **Zihan Cao:** validation (equal). **Ruiheng Yang:** funding acquisition (equal), validation (equal). **Ying Lin:** data curation (equal), investigation (equal), visualization (equal). **Ying Song:** data curation (equal). **Aiguo Xu:** funding acquisition (equal).

## Ethics Statement

This study does not involve any human or animal testing.

## Consent

Written informed consent was obtained from all study participants.

## Conflicts of Interest

The authors declare no conflicts of interest.

## Supporting information




**Figure S1:** Class Count Ring of the fruiting body of morels.

**Figure S2:** Venn diagram of differences among groups.

**Figure S3:** Difference multiples bar chart of metabolites.

**Figure S4:** Differential metabolite clustering heat map.

**Figure S5:** Difference multiples bar chart of metabolites.

**Table S1:** 1419 non‐volatile metabolites.

**Table S2:** 270 volatile metabolites.

**Table S3:** 920 significantly differential non‐volatile metabolites.

**Table S4:** 175 significantly differential volatile metabolites.

**Table S5:** Changes of non‐volatile metabolites with significant differences.

**Table S6:** Changes of volatile metabolites with significant differences.

## Data Availability

The original contributions presented in the study are included in the article/Supporting Information, further inquires can be directed to the corresponding author.

## References

[fsn370826-bib-0001] Ahmed, R. , N. ulain Hira , M. Wang , S. Iqbal , J. Yi , and Y. Hemar . 2023. “Genipin, a Natural Blue Colorant Precursor: Source, Extraction, Properties, and Applications.” Food Chemistry 137‐498: 137498. 10.1016/j.foodchem.2023.137498. 37741231

[fsn370826-bib-0002] Aisala, H. , J. Sola , A. Hopia , K. M. Linderborg , and M. Sandell . 2019. “Odor‐Contributing Volatile Compounds of Wild Edible Nordic Mushrooms0020Analyzed With HS–SPME–GC–MS and HS–SPME–GC–O/FID.” Food Chemistry 283: 566–578. 10.1016/j.foodchem.2019.01.053. 30722913

[fsn370826-bib-0003] Brancato, D. J. 1982. “Recognizing Potential Toxicity of Phenol.” Veterinary and Human Toxicology 24, no. 1: 29–30. 10.1007/BF02242168. 7036514

[fsn370826-bib-0004] Dang, Y. , Z. Liu , X. Gao , X. Gao , J. Cao , and G. Bao . 2016. “Determination of Volatiles in Ham by Gas Chromatography With Olfactory Detection.” International Journal of Food Engineering 12, no. 4: 323–332. 10.1515/ijfe-2015-0149.

[fsn370826-bib-0005] Davis, J. P. , and L. L. Dean . 2016. “Peanut Composition, Flavor and Nutrition ‐ Sciencedirect.” Peanuts: 289–345.

[fsn370826-bib-0006] Defraeye, T. 2017. “Impact of Size and Shape of Fresch‐Cut Fruit on the Drying Time and Fruit Quality.” Journal of Food Engineering 210, no. OCT: 35–41. 10.1016/j.jfoodeng.2017.04.004.

[fsn370826-bib-0007] Dong, H. , X. Zhao , M. Cai , et al. 2022. “Metabolomics Analysis of Morchella sp. From Different Geographical Origins of China Using UPLC‐Q‐TOF‐MS.” Frontiers in Nutrition 9: 865531. 10.3389/fnut.2022.865531. 35449541 PMC9016275

[fsn370826-bib-0008] Du, X. , J. Sissons , M. Shanks , and A. Plotto . 2021. “Aroma and Flavor Profile of Raw and Roasted Agaricus Bisporus Mushrooms Using a Panel Trained With Aroma Chemicals.” LWT 138: 110596. 10.1016/j.lwt.2020.110596.

[fsn370826-bib-0009] Du, X. H. , Q. Zhao , and Z. L. Yang . 2015. “A Review on Research Advances, Issues, and Perspectives of Morels.” Mycology 6, no. 2: 78–85. 10.1080/21501203.2015.1016561. 30151316 PMC6106076

[fsn370826-bib-0010] Erenturk, S. , M. S. Gulaboglu , and S. Gultekin . 2005. “The Effects of Cutting and Drying Medium on the Vitamin C Content of Rosehip During Drying.” Journal of Food Engineering 68, no. 4: 513–518. 10.1016/j.jfoodeng.2004.07.012.

[fsn370826-bib-0011] Gao, F. , W. Xie , H. Zhang , Z. Li , S. Li , and T. Li . 2022. “Metabolomic Analysis of Browning Mechanisms of Morels (Morchella Sextelata) During Storage.” Postharvest Biology and Technology 185: 111801. 10.1016/j.postharvbio.2021.111801.

[fsn370826-bib-0012] Guo, Y. , D. Chen , Y. Dong , H. Ju , C. Wu , and S. Lin . 2018. “Characteristic Volatiles Fingerprints and Changes of Volatile Compounds in Fresh and Dried Tricholoma Matsutake Singer by HS‐GC‐IMS and HS‐SPME‐GC–MS.” Journal of Chromatography B 1099: 46–55. 10.1016/j.jchromb.2018.09.011. 30241073

[fsn370826-bib-0013] Haq, F. U. , M. Imran , S. Saleem , A. Rafi , and M. Jamal . 2023. “Investigation of Three Morchella Species for Anticancer Activity Against Colon Cancer Cell Lines by UPLC‐MS‐Based Chemical Analysis.” Applied Biochemistry and Biotechnology 195, no. 1: 486–504. 10.1007/s12010-022-04131-z. 36094647

[fsn370826-bib-0014] Hiraide, M. , Y. Miyazaki , and Y. Shibata . 2004. “The Smell and Odorous Components of Dried Shiitake Mushroom, Lentinula Edodes I: Relationship Between Sensory Evaluations and Amounts of Odorous Components.” Journal of Wood Science 50: 358–364. 10.1007/s10086-003-0568-0.

[fsn370826-bib-0056] Hong, S. , K. K. Park , J. Magae , et al. 2005. “Ascochlorin Inhibits Matrix Metalloproteinase‐9 Expression by Suppressing Activator Protein‐1‐mediated Gene Expression Through the ERK1/2 Signaling Pathway.” Journal of Biological Chemistry 280, no. 26: 25202–25209. 15863510 10.1074/jbc.M413985200

[fsn370826-bib-0016] Hu, L. , J. Bi , X. Jin , Y. Qiu , and R. V. D. Sman . 2021. “Study on the Rehydration Quality Improvement of Shiitake Mushroom by Combined Drying Methods.” Food 10, no. 4: 769. 10.3390/foods10040769. PMC8065859 33916865

[fsn370826-bib-0017] Huang, X. l. , Y. h. Zhao , and Z. h. Hou . 2021. “Purification of Ethyl Linoleate From Foxtail Millet (*Setaria Italica*) Bran Oil via Urea Complexation and Molecular Distillation.” Food 10, no. 8: 1925–1925. 10.3390/foods10081925 PMC8392090 34441702

[fsn370826-bib-0018] Ito, Y. , and K. Sato . 2021. “Salicylamide Enhances Melanin Synthesis in B16F1 Melanoma Cells.” Biomolecules & Therapeutics 29, no. 4: 445–451. 10.4062/biomolther.2020.222. 33731492 PMC8255140

[fsn370826-bib-0019] Kim, Y. D. , J. H. Park , B. J. Park , M. J. In , D. C. Park , and N. S. Oh . 2014. “Combination Effect of ʟ‐Arginine and ʟ‐Aspartic Acid on Saltiness Enhancement of NaCl Solution.” Journal of Applied Biological Chemistry 57, no. 3: 251–254. 10.3839/jabc.2014.040.

[fsn370826-bib-0020] Ko, Y. C. , H. S. Choi , S. L. Kim , B. S. Yun , and D. S. Lee . 2022. “Anti‐Inflammatory Effects of (9Z, 11E)‐13‐Oxooctadeca‐9, 11‐Dienoic Acid (13‐KODE) Derived From *Salicornia Herbacea* L. on Lipopolysaccharide‐Stimulated Murine Macrophage via NF‐kB and MAPK Inhibition and Nrf2/HO‐1 Signaling Activation.” Antioxidants 11, no. 2: 180. 10.3390/antiox11020180. 35204063 PMC8868157

[fsn370826-bib-0021] Li, F. , Y. Jin , J. Wang , and H. Xu . 2022. “Structure Identification of Two Polysaccharides From Morchella Sextelata With Antioxidant Activity.” Food 11, no. 7: 982. 10.3390/foods11070982. PMC8997402 35407069

[fsn370826-bib-0022] Li, H. , L. Xie , Y. Ma , M. Zhang , Y. Zhao , and X. Zhao . 2019. “Effects of Drying Methods on Drying Characteristics, Physicochemical Properties and Antioxidant Capacity of Okra.” Lwt 101: 630–638. 10.1016/j.lwt.2018.11.076.

[fsn370826-bib-0023] Li, J. , H. Wu , Y. Liu , et al. 2021. “The Chemical Structure and Immunomodulatory Activity of an Exopolysaccharide Produced by Morchella Esculenta Under Submerged Fermentation.” Food & Function 12, no. 19: 9327–9338. 10.1039/d1fo01683k. 34606556

[fsn370826-bib-0024] Li, X. B. , Y. M. Zhang , C. E. Heng , et al. 2023. “Characteristic Fingerprints and Comparison of Volatile Flavor Compounds in Morchella Sextelata Under Different Drying Methods.” Food Research International 172: 113103–113103. 37689871 10.1016/j.foodres.2023.113103

[fsn370826-bib-0026] Liu, C. , Y. Sun , Q. Mao , et al. 2016. “Characteristics and Antitumor Activity of Morchella Esculenta Polysaccharide Extracted by Pulsed Electric Field.” International Journal of Molecular Sciences 17, no. 6: 986. 10.3390/ijms17060986. 27338370 PMC4926515

[fsn370826-bib-0027] Liu, J. R. , B. Q. Chen , B. F. Yang , et al. 2004. “Apoptosis of Human Gastric Adenocarcinoma Cells Induced by β‐Ionone.” World Journal of Gastroenterology 10, no. 3: 348. 10.3748/wjg.v10.i3.348. 14760755 PMC4724910

[fsn370826-bib-0028] Maga, J. A. 1981. “Mushroom Flavor.” Journal of Agricultural and Food Chemistry 29, no. 1: 1–4.

[fsn370826-bib-0029] Matsui, T. , C. Ito , M. Itoigawa , and T. Shibata . 2022. “Three Phlorotannins From *Sargassum carpophyllum* Are Effective Against the Secretion of Allergic Mediators From Antigen‐Stimulated Rat Basophilic Leukemia Cells.” Food Chemistry 377: 131992. 10.1016/j.foodchem.2021.131992. 34998157

[fsn370826-bib-0030] Mortel, E. L. M. V. D. , Z. A. Shen , J. F. Barnett , L. Krsmanovic , A. Myhre , and B. F. Delaney . 2010. “Afety Assessment of n‐Acetyl‐l‐Threonine.” Food and Chemical Toxicology 48, no. 7: 1919–1925. 10.1016/j.fct.2010.04.035. 20434501

[fsn370826-bib-0031] Mulet, A. , J. Garcia‐Reverter , J. Bon , and A. Berna . 2000. “Effect of Shape on Potato and Cauliflower Shrinkage During Drying.” Drying Technology 18, no. 6: 1201–1219. 10.1080/07373930008917772.

[fsn370826-bib-0032] Nooshkam, M. , M. Varidi , and M. Bashash . 2019. “The Maillard Reaction Products as Food‐Born Antioxidant and Antibrowning Agents in Model and Real Food Systems.” Food Chemistry 275: 644–660. 10.1016/j.foodchem.2018.09.083. 30724245

[fsn370826-bib-0033] Qin, L. , J. X. Gao , J. Xue , et al. 2020. “Changes in Aroma Profile of Shiitake Mushroom (Lentinus Edodes) During Different Stages of Hot Air Drying.” Food 9, no. 4: 444. 10.3390/foods9040444. PMC7230619 32272549

[fsn370826-bib-0034] Shen, Q. , Z. He , Y. Ding , and L. Sun . 2023. “Effect of Different Drying Methods on the Quality and Nonvolatile Flavor Components of Oudemansiella Raphanipes.” Food 12, no. 3: 676. 10.3390/foods12030676. PMC9914412 36766204

[fsn370826-bib-0035] Shi, J. , S. Wang , R. Tong , et al. 2022. “Widely Targeted Secondary Metabolomics Explored Pomegranate Aril Browning During Cold Storage.” Postharvest Biology and Technology 186: 111839. 10.1016/j.postharvbio.2022.111839.

[fsn370826-bib-0036] Sun, L. B. , Z. Y. Zhang , G. Xin , et al. 2020. “Advances in Umami Taste and Aroma of Edible Mushrooms.” Trends in Food Science & Technology 96: 176–187. 10.1016/j.tifs.2019.12.018.

[fsn370826-bib-0037] Sunil, C. , and B. Xu . 2022. “Mycochemical Profile and Health‐Promoting Effects of Morel Mushroom Morchella Esculenta (L.)–a Review.” Food Research International 159: 111571. 10.1016/j.foodres.2022.111571. 35940783

[fsn370826-bib-0038] Tietel, Z. , and S. Masaphy . 2018a. “Aroma‐Volatile Profile of Black Morel (Morchella Importuna) Grown in Israel.” Journal of the Science of Food and Agriculture 98, no. 1: 346–353. 10.1002/jsfa.8477. 28597472

[fsn370826-bib-0039] Tietel, Z. , and S. Masaphy . 2018b. “True Morels (Morchella)—Nutritional and Phytochemical Composition, Health Benefits and Flavor: A Review.” Critical Reviews in Food Science and Nutrition 58, no. 11: 1888–1901. 10.1080/10408398.2017.1285269. 28350213

[fsn370826-bib-0040] Tietel, Z. , and S. Masaphy . 2022. “Chemotyping of Three Morchella Species Reveals Species‐and Age‐Related Aroma Volatile Biomarkers.” Lwt 154: 112587. 10.1016/j.lwt.2021.112587.

[fsn370826-bib-0041] Tolera, K. D. , and S. Abera . 2017. “Nutritional Quality of Oyster Mushroom (Pleurotus Ostreatus) as Affected by Osmotic Pretreatments and Drying Methods.” Food Science & Nutrition 5, no. 5: 989–996. 10.1002/fsn3.484. 28948016 PMC5608979

[fsn370826-bib-0042] Uawisetwathana, U. , and N. Karoonuthaisiri . 2019. “Metabolomics for Rice Quality and Traceability: Feasibility and Future Aspects.” Current Opinion in Food Science 28: 58–66. 10.1016/j.cofs.2019.08.008.

[fsn370826-bib-0043] Wang, H. , J. Hua , Q. Yu , et al. 2021. “Widely Targeted Metabolomic Analysis Reveals Dynamic Changes in Non‐Volatile and Volatile Metabolites During Green Tea Processing.” Food Chemistry 363: 130131. 10.1016/j.foodchem.2021.130131. 34120048

[fsn370826-bib-0044] Wang, H. Y. , H. Qian , and W. R. Yao . 2011. “Melanoidins Produced by the Maillard Reaction: Structure and Biological Activity.” Food Chemistry 128, no. 3: 573–584. 10.1016/j.foodchem.2011.03.075.

[fsn370826-bib-0045] Wang, Z. , H. Wang , Z. Kang , Y. Wu , Y. Xing , and Y. Yang . 2020. “Antioxidant and Anti‐Tumour Activity of Triterpenoid Compounds Isolated From Morchella Mycelium.” Archives of Microbiology 202: 1677–1685. 10.1007/s00203-020-01876-1. 32285166

[fsn370826-bib-0046] Wen, X. , W. Li , W. Li , et al. 2022. “Quality Characteristics and Non‐Volatile Taste Formation Mechanism of Lentinula Edodes During Hot Air Drying.” Food Chemistry 393: 133378. 10.1016/j.foodchem.2022.133378. 35667179

[fsn370826-bib-0047] Woo, J. H. , D. Y. Nam , H. J. Kim , P. T. L. Hong , and J. H. Nam . 2021. “Nootkatol Prevents Ultraviolet Radiation‐Induced Photoaging via orai1 and trpv1 Inhibition in Melanocytes and Keratinocytes.” Korean Journal of Physiology & Pharmacology 25, no. 1: 87–94. 10.4196/kjpp.2021.25.1.87. 33361541 PMC7756533

[fsn370826-bib-0048] Xiao, Y. , C. He , Y. Chen , et al. 2022. “UPLC–QQQ–MS/MS‐Based Widely Targeted Metabolomic Analysis Reveals the Effect of Solid‐State Fermentation With Eurotium Cristatum on the Dynamic Changes in the Metabolite Profile of Dark Tea.” Food Chemistry 378: 131999. 10.1016/j.foodchem.2021.131999. 35081481

[fsn370826-bib-0049] Yu, H. , R. Zhang , F. Yang , et al. 2021. “Control Strategies of Pyrazines Generation From Maillard Reaction.” Trends in Food Science & Technology 112: 795–807. 10.1016/j.tifs.2021.04.028.

[fsn370826-bib-0050] Zhang, H. , D. Pu , B. Sun , F. Ren , Y. Zhang , and H. Chen . 2018. “Characterization and Comparison of Key Aroma Compounds in Raw and Dry Porcini Mushroom (Boletus Edulis) by Aroma Extract Dilution Analysis, Quantitation and Aroma Recombination Experiments.” Food Chemistry 258: 260–268. 10.1016/j.foodchem.2018.03.056. 29655732

[fsn370826-bib-0051] Zhang, L. , J. Chen , J. Zhang , et al. 2022. “Lipid Oxidation in Fragrant Rapeseed Oil: Impact of Seed Roasting on the Generation of Key Volatile Compounds.” Food Chemistry 16: 100491. 10.1016/j.fochx.2022.100491. PMC9626899 36339322

[fsn370826-bib-0052] Zhang, L. , X. Dong , X. Feng , S. A. Ibrahim , W. Huang , and Y. Liu . 2021. “Effects of Drying Process on the Volatile and Non‐Volatile Flavor Compounds of Lentinula Edodes.” Food 10, no. 11: 2836. 10.3390/foods10112836. PMC8622265 34829114

[fsn370826-bib-0053] Zhang, M. , S. Xing , C. Fu , et al. 2022. “Effects of Drying Methods on Taste Components and Flavor Characterization of Cordyceps Militaris.” Food 11, no. 23: 3933. 10.3390/foods11233933. PMC9735880 36496741

[fsn370826-bib-0054] Zhou, X. M. , C. J. Zheng , J. T. Wu , G. Y. Chen , J. Chen , and C. G. Sun . 2016. “Five New Lactone Derivatives From the Stems of Dendrobium Nobile.” Fitoterapia 115: 96–100. 10.1016/j.fitote.2016.10.002. 27720924

[fsn370826-bib-0055] Zou, S. , J. Wu , M. Q. Shahid , et al. 2020. “Identification of Key Taste Components in Loquat Using Widely Targeted Metabolomics.” Food Chemistry 323: 126822. 10.1016/j.foodchem.2020.126822. 32334307

