# Supplementary figures and images for: Widely Targeted Metabolomics of Morchella Sextelata After Hot‐Air Drying
Source: Food Sci Nutr. 2025 Sep 18;13(9):e70826. doi: 10.1002/fsn3.70826 (PMC12445116; doi:10.1002/fsn3.70826)

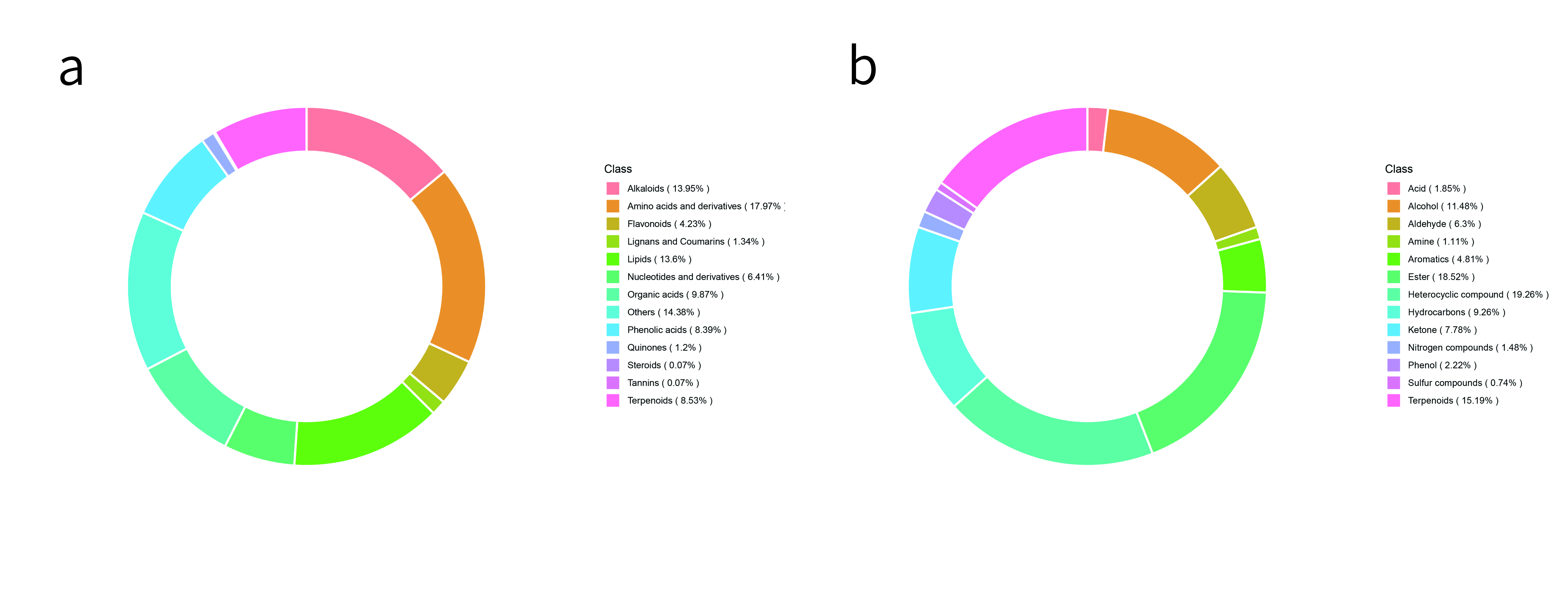

Supplement: Supplementary file 1 — Figure S1: Class Count Ring of the fruiting body of morels. [file FSN3-13-e70826-s009.tif]

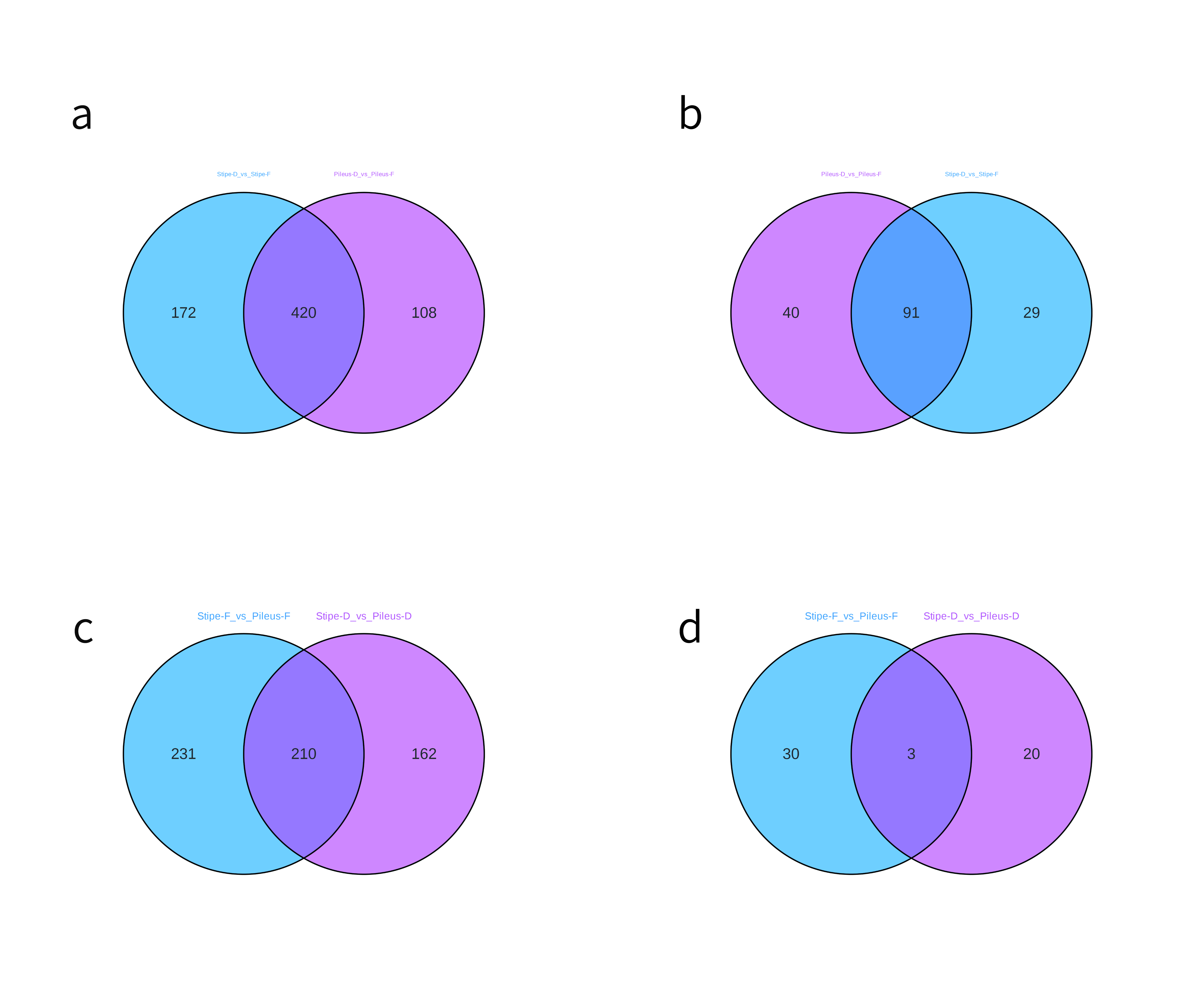

Supplement: Supplementary file 2 — Figure S2: Venn diagram of differences among groups. [file FSN3-13-e70826-s010.tif]

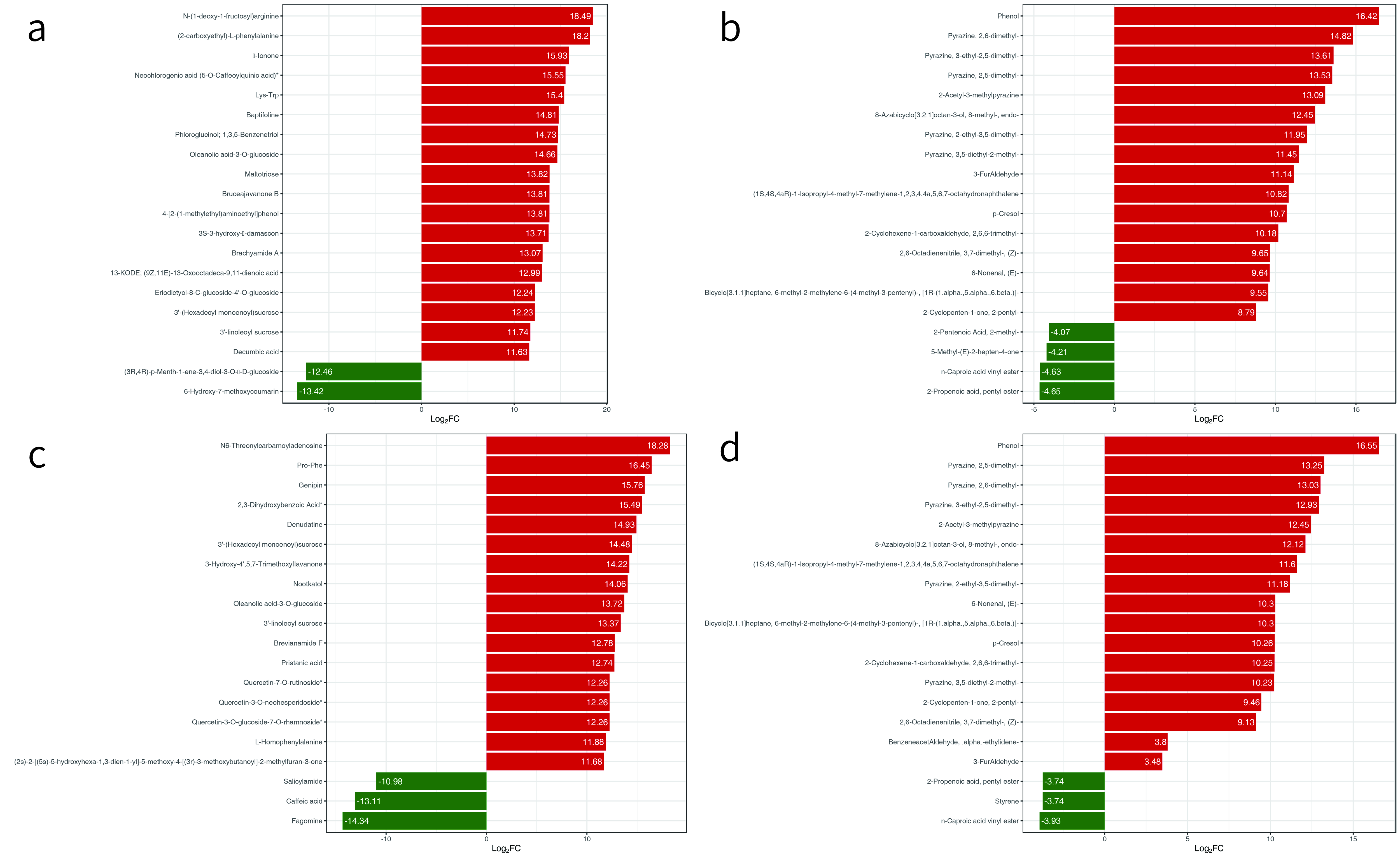

Supplement: Supplementary file 3 — Figure S3: Difference multiples bar chart of metabolites. [file FSN3-13-e70826-s002.tif]

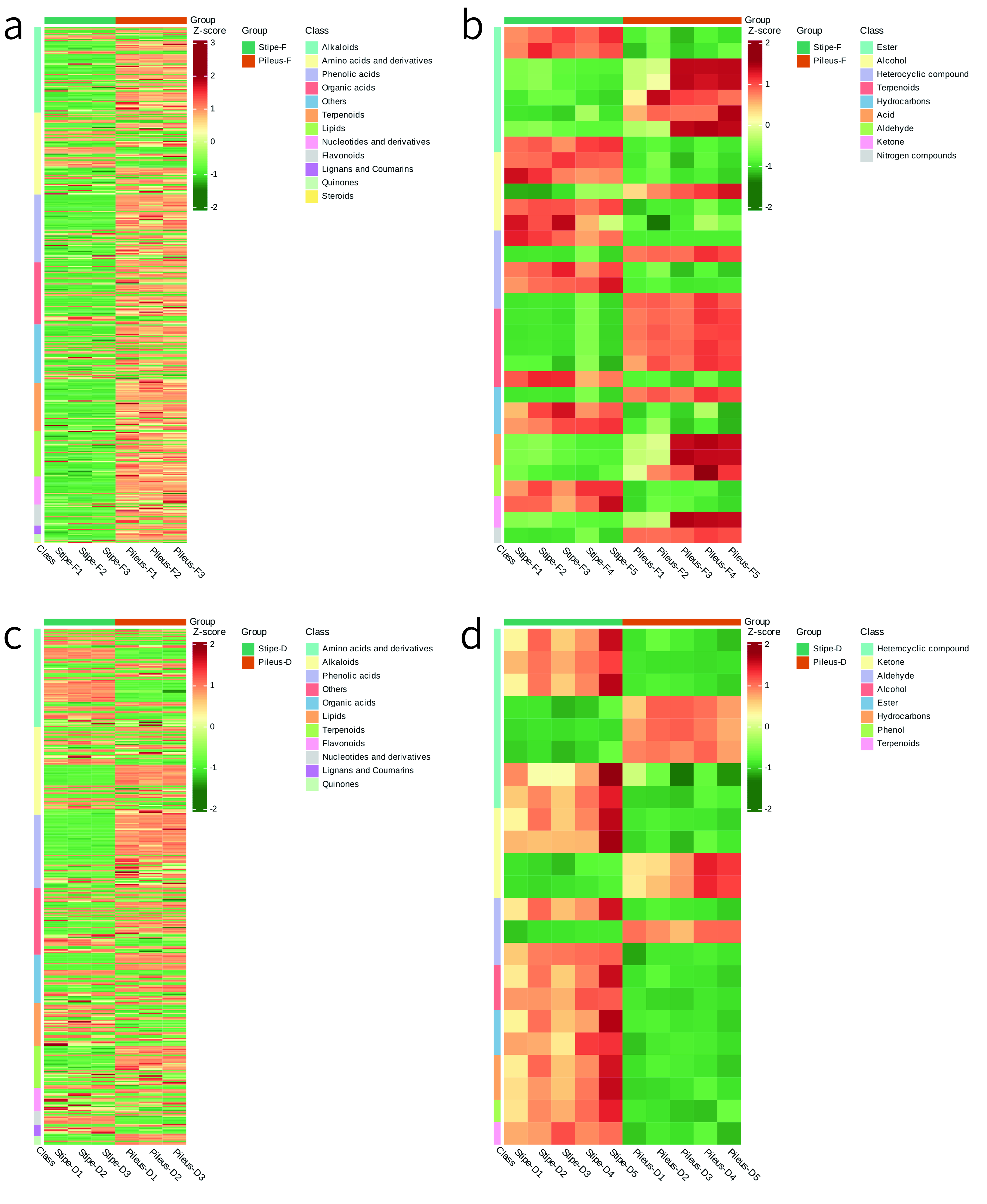

Supplement: Supplementary file 4 — Figure S4: Differential metabolite clustering heat map. [file FSN3-13-e70826-s003.tif]

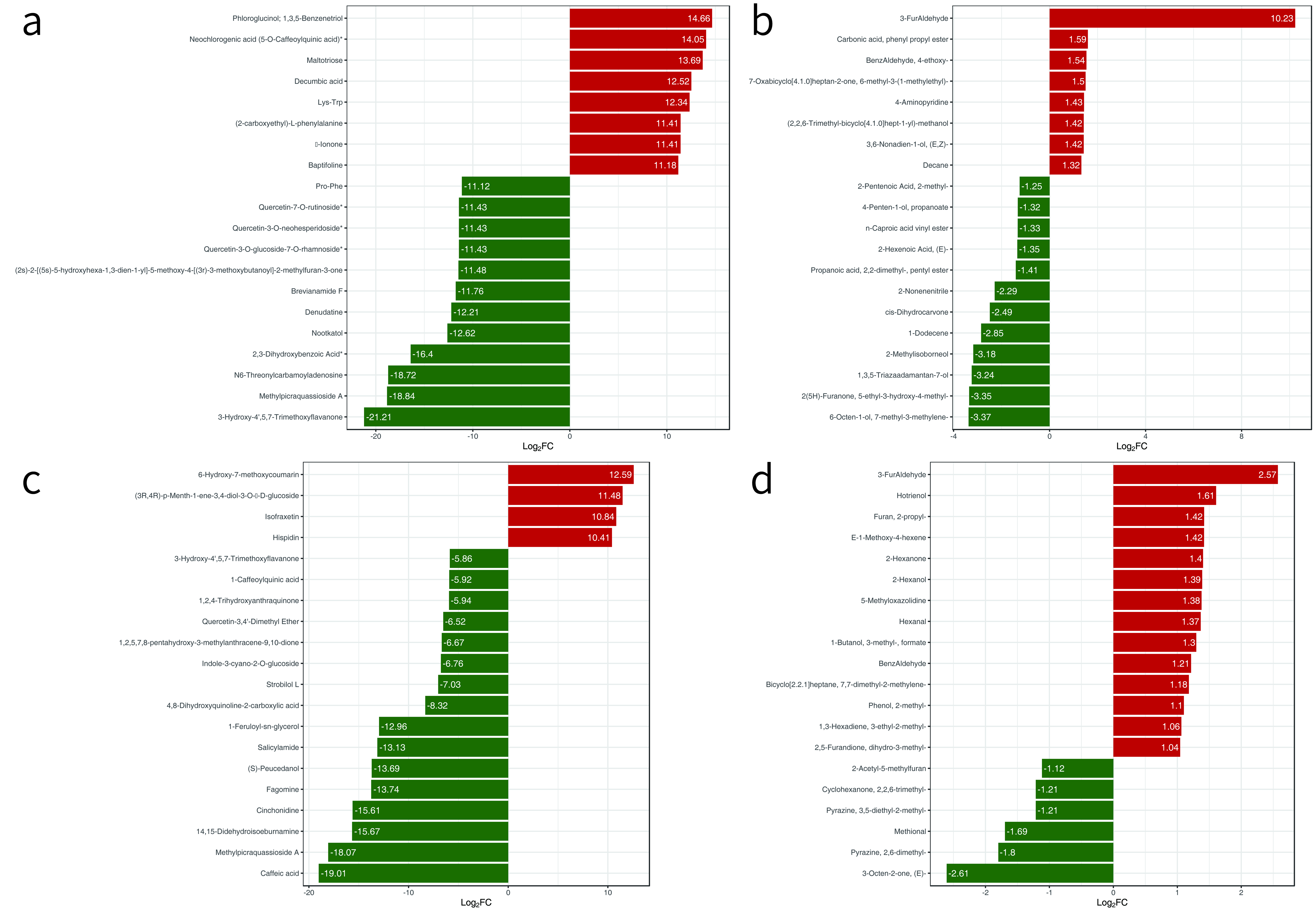

Supplement: Supplementary file 5 — Figure S5: Difference multiples bar chart of metabolites. [file FSN3-13-e70826-s008.tif]
